# Supplementary material for: Identification of a Gene Signature That Predicts Dependence upon YAP/TAZ-TEAD
Source: Cancers (Basel). 2024 Feb 20;16(5):852. doi: 10.3390/cancers16050852 (PMC10930532; doi:10.3390/cancers16050852)
Supplement: Supplementary file 1 [file cancers-16-00852-s001.zip › cancers-2821103-supplementary final.pdf]

Supplemental Figure and Table Legends:

Figure S1. DepMap dependency scores for melanoma cell lines. Plots downloaded directly from DepMap showing Dependency Scores (CRISPR Public23Q2+Score, Chronos) and log2(TPM+1) expression for *YAP1*, *WWTR1*, or *TEAD1*. Cell lines with a Chronos Dependency Score of $\leq$ -0.65 are colored in red. The dependency scores and expression data for each cell line are listed in Table S4, Tab 1.

Figure S2. *YAP* and *WWTR1* mRNA expression do not strongly correlate with the mRNA expression of their target genes. RNA-seq data from the TCGA-SKCM project were downloaded and analyzed. (**A**) X-Y plots show the TPM value for the indicated gene for each individual tumor (dots). Correlation analysis was run for each comparison and the Pearson Correlation Coefficient (r) is indicated on the plots. (**B**) TCGA-SKCM tumors with high ($\geq$ 1 standard deviation from the mean) or low ($\leq$ -1 standard deviation from the mean) expression of *YAP*1, *WWTR1*, or *CTGF* (**B**) were analyzed for the expression of each indicated gene for each individual tumor (dots). Mean $\pm$ S.E.M. is shown. Statistical significance was determined using two-tail unpaired t-test; ** $p \leq 0.01$, **** $p \leq 0.0001$, n.s. $p>0.05$. (**C**) GSEA was run on the TCGA-SKCM RNA-seq data to test for the enrichment of our YAP/TAZ Up geneset in *CTGF* or *CYR61* high vs. low tumors. The data used to generate this figure can be found in Table S5.

Figure S3. Downregulated YAP/TAZ signature genes are not negatively correlated with YAP/TAZ activity in human melanomas. RNA-seq data from the TCGA-SKCM project were downloaded and tumors with high ($\geq$ 1 standard deviation from the mean) or low ($\leq$ -1 standard deviation from the mean) expression of *CTGF* (**A**) or *CYR61* mRNA (**B**) were analyzed. (**A,B**) GSEA analysis was performed to test for the enrichment of our YAP/TAZ Down geneset in *CTGF* or *CYR61* high vs. low tumors (NES and FDR are indicated). The plots show the relative expression (Z-Score of the log transformed TPM (log2(1+TPM))) of each of the 52 YAP/TAZ Down genes in *CTGF* (**A**) or *CYR61* (**B**) high vs. low tumors. The tumors are sorted by *CTGF* or *CYR61* mRNA expression and genes are ranked from highest (top) to lowest (bottom) based on the Spearman Rank Correlation of the gene with either *CTGF* (**A**) or *CYR61* (**B**). (**C**) The heatmap shows the Spearman Rank Correlation values for each gene compared to *CTGF* and *CYR61*. The data used to generate this figure can be found in Table S5.

Table S1. Reagents and datasets used in this study. (**Tab 1**) The top table shows existing, purchased, or gifted vectors used in this study and their source. The bottom table shows all new vectors that were cloned for this study and lists the source vector for the insert and the backbone. For new mutant constructs, the insert that was mutated is listed. The publications that describe each vector are indicated [29-32]. (**Tab 2**) A list of qPCR primers used in this study. (**Tab 3**) The list of publicly available gene expression datasets used in this study. The GEO accession number for each dataset, cell line name, species, type of cell line, comparison made, and the citation with Pubmed ID are listed for each dataset [6,44,46,50-68]. (**Tab 4**) A list of YAP, YAP/TAZ, and Hippo Pathway genesets used in this study and their source [47-49]. Most were downloaded directly from the Molecular Signature Database [39] . (**Tab 5**) The list of ENCODE datasets used in this study with ENCODE experiment names and numbers, Conservative IDR Threshold Peaks, " .Bed" Data file accession numbers, and the TEAD that was immunoprecipitated indicated.

Table S2. Processed A375 RNA-seq data. Tables include RNA-seq data from A375 cells expressing control empty vector or the indicated YAP constructs. (**Tab 1**) The entire dataset with the log2 fragments per kilobase million (l2FPKM) for each replicate and the log2FC, adjusted pValue and qValue for each gene for the indicated comparisons. (**Tab 2**) A subset of the data in Tab 1 that includes only differentially expressed genes (DEG) (fold change > 2, adjusted pValue < 0.05) for each comparison. (**Tabs 3,4**) The lists of up and downregulated genes in each comparison. "1" indicates the gene was differentially expressed (fold change > 2, adjusted pValue < 0.05) in that comparison and an empty cell indicates that it was not.

Table S3. Lists of YAP/TAZ-regulated genes in metastatic melanoma cell lines. (**Tab 1**) Differentially expressed genes (DEG) (fold change > 2, adjusted pValue < 0.05) in A375-YAP^{2SA} vs. Control cells. (**Tabs 2,3**) The GSE68599 array dataset [44] was analyzed in ExAtlas to identify genes up or downregulated in siControl vs. siYAP/siTAZ-transfected SK-MEL-28 or WM3248 cells (fold change > 2, FDR$\leq$0.05). (**Tab 4**) The list of up or downregulated genes in MeWo cells expressing YAP^{5SA} vs. Control (Zhang et al. 2020). (**Tab 5**) The list of all up or down regulated genes from Tabs 1-4. Thess data were used to generate the Venn diagram in Figure 2A. (**Tab 6**) A YAP/TAZ gene signature consisting of genes up- or

downregulated in A375-YAP[2SA] cells and at least 2 of the 3 other melanoma cell lines. Included are the log2FC values from Tabs 1-4. These data were used to generate the heatmap in Figure 2C.

Table S4. DepMap data. RNA-seq for protein coding genes and Dependency Scores (CRISPR Public23Q2+Score, Chronos) for *YAP1*, *WWTR1* (TAZ), and *TEADs1-4* that were downloaded from the DepMap portal are provided here. (**Tab 1**) Dependency Scores (CRISPR Public23Q2+Score, Chronos) for *YAP1*, *WWTR1*, and *TEADs1-4* for each melanoma cell line in the DepMap dataset. (**Tab 2**) RNA-seq data for each melanoma cell line downloaded from DepMap (values are log2(TPM+1)). (**Tab 3**) Melanoma cell line Dependency Scores and RNA-seq data (TPM) from Tabs 1 and 2 for all 132 genes in our YAP/TAZ signature. Green genes are in the YAP/TAZ Up geneset and red genes are in the YAP/TAZ Down geneset. Cell lines are organized based on Dependency Scores for *TEAD1*, *WWTR1,* and then *YAP1* with yellow cells highlighting dependent lines. Dependency scores were used to generate the heatmap in Figure 5B, top. (**Tab 4**) RNA-seq data for each YAP/TAZ Up gene in the melanoma cell lines were Z-Scored and then the Spearman's Rank Correlation of each gene with *CTGF* was calculated. Spearman's Rank Correlation values (column B) were used to sort genes (highest to lowest). The Z-Scored expression data were used for the heatmap in Figure 5B bottom. (**Tab 5**) The GSVA scores for the indicated genesets in each melanoma cell line analyzed in Figure 5C. Whether the cell line was scored as YAP, TAZ, YAP/TAZ, or TEAD1 dependent (based on DepMap Chronos Dependency Score) is indicated. These data were used to generate the ROC curves in Figure 5C.

Table S5. TCGA Data. RNA-seq data from human melanoma samples in the TCGA-SKCM project were downloaded and processed. Tumors with high ($\geq$1 standard deviation from the mean) and low ($\leq$ -1 standard deviation from the mean) expression of *YAP1*, *WWTR1*, *CTGF*, and *CYR61* were identified. (**Tabs 1-4**) The expression (TPM) of each gene in our YAP/TAZ signature in tumors with high and low expression of *YAP1* (**Tab 1**), *WWTR1* (**Tab 2**), *CTGF* (**Tab 3**), and *CYR61* (**Tab 4**). The data in Tabs 3 and 4 were used to generate the heatmaps in Figures 6B,E and Figure S3A,B. **(Tab 5**) The Spearman Rank Correlation value for each pairwise comparison of genes in the *CTGF* high and low tumors from Tab 3. These data were used to generate the heatmap in Figure 6C. (**Tab 6**) The Spearman Rank Correlation value for each pairwise comparison of genes in the *CYR61* high and low tumors from Tab 4. These data were used to generate the heatmap in Figure 6F. (**Tab 7**) The Spearman Rank Correlation values for each gene compared to *YAP1*, *WWTR1*, *CTGF*, and *CYR61*. Genes with correlation values $\geq$ 0.4 are indicated in blue and those between 0.39 and 0.3 in yellow. Some of these data were used to generate the heatmaps in Figure 6G,H and Figure S3C.

Table S6. YAP/TAZ signature genes are YAP/TAZ-responsive in other cell lines. (**Tab 1**) GSEA was used to generate a rank-ordered list for the indicated comparison in each cell line. For each comparison the % Rank (the gene's rank/total genes in rank-ordered list*100) of each of the 132 genes in our YAP/TAZ signature is shown. Green genes are in the YAP/TAZ Up geneset and red genes are in the YAP/TAZ Down geneset. These data were used to generate the heatmap in Figure 7A. (**Tab 2**) The lists of genes that overlap between the YAP Up and YAP/TAZ Up genesets depicted in the Venn Diagram in Figure 7B. (**Tab 3**) GSEA was performed on the indicated datasets and comparisons using the indicated genesets. The table lists the Normalized Enrichment Score (NES), False Discovery Rate (FDR), and % of each geneset that was in the Leading Edge (% Leading Edge) for Phenotype #1. A positive NES indicates the geneset was enriched in Phenotype #1 and a negative NES indicates the geneset was enriched in Phenotype #2. If a geneset was not enriched, there is no leading edge for that geneset, which is indicated by an "x". These data were used to generate the heatmaps in Figure 7C. (**Tab 4**) The list indicates which of the 78 genes from our YAP/TAZ Up geneset were determined to be in the leading edge of the GSEA analysis found in Figure 7D. "Yes" indicates the gene was in the leading edge for the indicated analysis. (**Tab 5**) The GSVA scores for the indicated genesets in each of the 1019 cell lines from the DepMap database that we analyzed. Whether the cell line was scored as YAP, TAZ, YAP/TAZ, or TEAD dependent (based on DepMap Chronos Dependency Score) is indicated. These data were used to generate the ROC curves in Figure 7E.

Figure S1. DepMap dependency scores for melanoma cell lines. Plots downloaded directly from DepMap showing Dependency Scores (CRISPR Public23Q2+Score, Chronos) and log2(TPM+1) expression for *YAP1, WWTR1,* or *TEAD1.* Cell lines with a Chronos Dependency Score of $\leq$ -0.65 are colored in red. The dependency scores and expression data for each cell line are listed in Table S4, Tab 1.

Figure S2. *YAP* and *WWTR1* mRNA expression do not strongly correlate with the mRNA expression of their target genes. RNA-seq data from the TCGA-SKCM project were downloaded and analyzed. (**A**) X-Y plots show the TPM value for the indicated gene for each individual tumor (dots). Correlation analysis was run for each comparison and the Pearson Correlation Coefficient (r) is

indicated on the plots. (**B**) TCGA-SKCM tumors with high (≥ 1 standard deviation from the mean) or low (≤ -1 standard deviation from the mean) expression of *YAP*1, *WWTR1*, or *CTGF* (**B**) were analyzed for the expression of each indicated gene for each individual tumor (dots). Mean ± S.E.M. is shown. Statistical significance was determined using two-tail unpaired t-test; ** p ≤ 0.01, **** p ≤ 0.0001, n.s. p>0.05. (**C**) GSEA was run on the TCGA-SKCM RNA-seq data to test for the enrichment of our YAP/TAZ Up geneset in *CTGF* or *CYR61* high vs. low tumors. The data used to generate this figure can be found in Table S5.

Figure S3. Downregulated YAP/TAZ signature genes are not negatively correlated with YAP/TAZ activity in human melanomas. RNA-seq data from the TCGA-SKCM project were downloaded and tumors with high (≥ 1 standard deviation from the mean) or low (≤ -1 standard deviation from the mean) expression of *CTGF* (**A**) or *CYR61* mRNA (**B**) were analyzed. (**A,B**) GSEA analysis was performed to test for the enrichment of our YAP/TAZ Down geneset in *CTGF* or *CYR61* high vs. low tumors (NES and FDR are indicated). The plots show the relative expression (Z-Score of the log transformed TPM (log2(1+TPM))) of each of the 52 YAP/TAZ Down genes in *CTGF* (**A**) or *CYR61* (**B**) high vs. low tumors. The tumors are sorted by *CTGF* or *CYR61* mRNA expression and genes are ranked from highest (top) to lowest (bottom) based on the Spearman Rank Correlation of the gene with either *CTGF* (**A**) or *CYR61* (**B**). (**C**) The heatmap shows the Spearman Rank Correlation values for each gene compared to *CTGF* and *CYR61*. The data used to generate this figure can be found in Table S5.

Table S1. Reagents and datasets used in this study. (**Tab 1**) The top table shows existing, purchased, or gifted vectors used in this study and their source. The bottom table shows all new vectors that were cloned for this study and lists the source vector for the insert and the backbone. For new mutant constructs, the insert that was mutated is listed. The publications that describe each vector are indicated [29-32]. (**Tab 2**) A list of qPCR primers used in this study. (**Tab 3**) The list of publicly available gene expression datasets used in this study. The GEO accession number for each dataset, cell line name, species, type of cell line, comparison made, and the citation with Pubmed ID are listed for each dataset [6,44,46,50-68]. (**Tab 4**) A list of YAP, YAP/TAZ, and Hippo Pathway genesets used in this study and their source [47-49]. Most were downloaded directly from the Molecular Signature Database [39] . (**Tab 5**) The list of ENCODE datasets used in this study with ENCODE experiment names and numbers, Conservative IDR Threshold Peaks, " .Bed” Data file accession numbers, and the TEAD that was immunoprecipitated indicated.

Table S2. Processed A375 RNA-seq data. Tables include RNA-seq data from A375 cells expressing control empty vector or the indicated YAP constructs. (**Tab 1**) The entire dataset with the log2 fragments per kilobase million (l2FPKM) for each replicate and the log2FC, adjusted pValue and qValue for each gene for the indicated comparisons. (**Tab 2**) A subset of the data in Tab 1 that includes only differentially expressed genes (DEG) (fold change > 2, adjusted pValue < 0.05) for each comparison. (**Tabs 3,4**) The lists of up and downregulated genes in each comparison. “1” indicates the gene was differentially expressed (fold change > 2, adjusted pValue < 0.05) in that comparison and an empty cell indicates that it was not.

Table S3. Lists of YAP/TAZ-regulated genes in metastatic melanoma cell lines. (**Tab 1**) Differentially expressed genes (DEG) (fold change > 2, adjusted pValue < 0.05) in A375-YAP^{2SA} vs. Control cells. (**Tabs 2,3**) The GSE68599 array dataset [44] was analyzed in ExAtlas to identify genes up or downregulated in siControl vs. siYAP/siTAZ-transfected SK-MEL-28 or WM3248 cells (fold change > 2, FDR≤0.05). (**Tab 4**) The list of up or downregulated genes in MeWo cells expressing YAP^{5SA} vs. Control (Zhang et al. 2020). (**Tab 5**) The list of all up or down regulated genes from Tabs 1-4. Thess data were used to generate the Venn diagram in Figure 2A. (**Tab 6**) A YAP/TAZ gene signature consisting of genes up- or downregulated in A375-YAP^{2SA} cells and at least 2 of the 3 other melanoma cell lines. Included are the log2FC values from Tabs 1-4. These data were used to generate the heatmap in Figure 2C.

Table S4. DepMap data. RNA-seq for protein coding genes and Dependency Scores (CRISPR Public23Q2+Score, Chronos) for *YAP1*, *WWTR1* (TAZ), and *TEADs1-4* that were downloaded from the DepMap portal are provided here. (**Tab 1**) Dependency Scores (CRISPR Public23Q2+Score, Chronos) for *YAP1*, *WWTR1*, and *TEADs1-4* for each melanoma cell line in the DepMap dataset. (**Tab 2**) RNA-seq data for each melanoma cell line downloaded from DepMap (values are log2(TPM+1)). (**Tab 3**) Melanoma cell line Dependency Scores and RNA-seq data (TPM) from Tabs 1 and 2 for all 132 genes in our YAP/TAZ signature. Green genes are in the YAP/TAZ Up geneset and red genes are in the YAP/TAZ Down geneset. Cell lines are organized based on Dependency

Scores for *TEAD1*, *WWTR1*, and then *YAP1* with yellow cells highlighting dependent lines. Dependency scores were used to generate the heatmap in Figure 5B, top. (**Tab 4**) RNA-seq data for each YAP/TAZ Up gene in the melanoma cell lines were Z-Scored and then the Spearman's Rank Correlation of each gene with *CTGF* was calculated. Spearman's Rank Correlation values (column B) were used to sort genes (highest to lowest). The Z-Scored expression data were used for the heatmap in Figure 5B bottom. (**Tab 5**) The GSVA scores for the indicated genesets in each melanoma cell line analyzed in Figure 5C. Whether the cell line was scored as YAP, TAZ, YAP/TAZ, or TEAD1 dependent (based on DepMap Chronos Dependency Score) is indicated. These data were used to generate the ROC curves in Figure 5C.

Table S5. TCGA Data. RNA-seq data from human melanoma samples in the TCGA-SKCM project were downloaded and processed. Tumors with high (≥1 standard deviation from the mean) and low (≤ -1 standard deviation from the mean) expression of *YAP1*, *WWTR1*, *CTGF*, and *CYR61* were identified. (**Tabs 1-4**) The expression (TPM) of each gene in our YAP/TAZ signature in tumors with high and low expression of *YAP1* (**Tab 1**), *WWTR1* (**Tab 2**), *CTGF* (**Tab 3**), and *CYR61* (**Tab 4**). The data in Tabs 3 and 4 were used to generate the heatmaps in Figures 6B,E and Figure S3A,B. **(Tab 5**) The Spearman Rank Correlation value for each pairwise comparison of genes in the *CTGF* high and low tumors from Tab 3. These data were used to generate the heatmap in Figure 6C. (**Tab 6**) The Spearman Rank Correlation value for each pairwise comparison of genes in the *CYR61* high and low tumors from Tab 4. These data were used to generate the heatmap in Figure 6F. (**Tab 7**) The Spearman Rank Correlation values for each gene compared to *YAP1*, *WWTR1*, *CTGF*, and *CYR61*. Genes with correlation values ≥ 0.4 are indicated in blue and those between 0.39 and 0.3 in yellow. Some of these data were used to generate the heatmaps in Figure 6G,H and Figure S3C.

Table S6. YAP/TAZ signature genes are YAP/TAZ-responsive in other cell lines. (**Tab 1**) GSEA was used to generate a rank-ordered list for the indicated comparison in each cell line. For each comparison the % Rank (the gene's rank/total genes in rank-ordered list*100) of each of the 132 genes in our YAP/TAZ signature is shown. Green genes are in the YAP/TAZ Up geneset and red genes are in the YAP/TAZ Down geneset. These data were used to generate the heatmap in Figure 7A. (**Tab 2**) The lists of genes that overlap between the YAP Up and YAP/TAZ Up genesets depicted in the Venn Diagram in Figure 7B. (**Tab 3**) GSEA was performed on the indicated datasets and comparisons using the indicated genesets. The table lists the Normalized Enrichment Score (NES), False Discovery Rate (FDR), and % of each geneset that was in the Leading Edge (% Leading Edge) for Phenotype #1. A positive NES indicates the geneset was enriched in Phenotype #1 and a negative NES indicates the geneset was enriched in Phenotype #2. If a geneset was not enriched, there is no leading edge for that geneset, which is indicated by an "x". These data were used to generate the heatmaps in Figure 7C. (**Tab 4**) The list indicates which of the 78 genes from our YAP/TAZ Up geneset were determined to be in the leading edge of the GSEA analysis found in Figure 7D. "Yes" indicates the gene was in the leading edge for the indicated analysis. (**Tab 5**) The GSVA scores for the indicated genesets in each of the 1019 cell lines from the DepMap database that we analyzed. Whether the cell line was scored as YAP, TAZ, YAP/TAZ, or TEAD dependent (based on DepMap Chronos Dependency Score) is indicated. These data were used to generate the ROC curves in Figure 7E.
