# Supplementary figures and images for: Identification of a Gene Signature That Predicts Dependence upon YAP/TAZ-TEAD
Source: Cancers (Basel). 2024 Feb 20;16(5):852. doi: 10.3390/cancers16050852 (PMC10930532; doi:10.3390/cancers16050852)

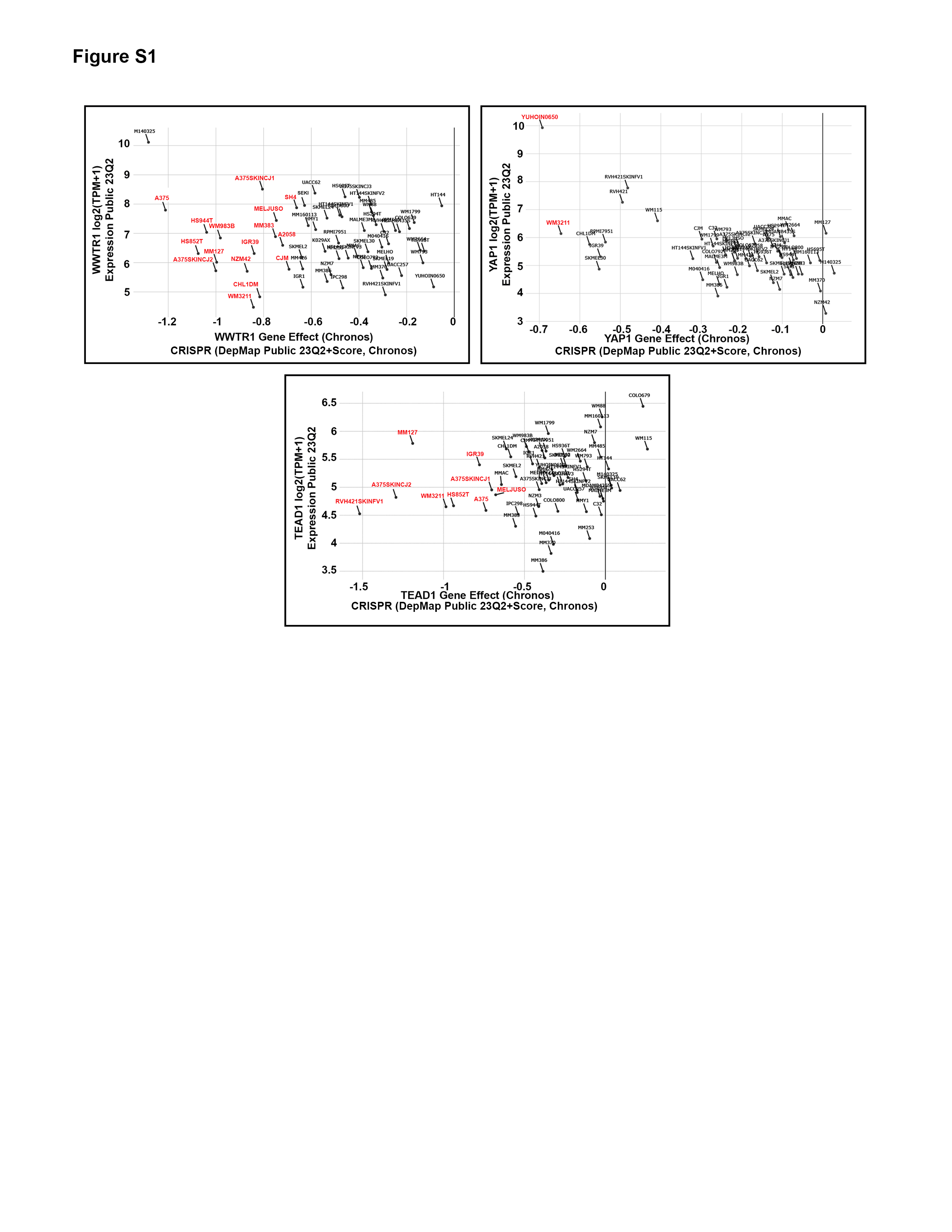

Supplement: Supplementary file 1 [file cancers-16-00852-s001.zip › Figure S1.tif]

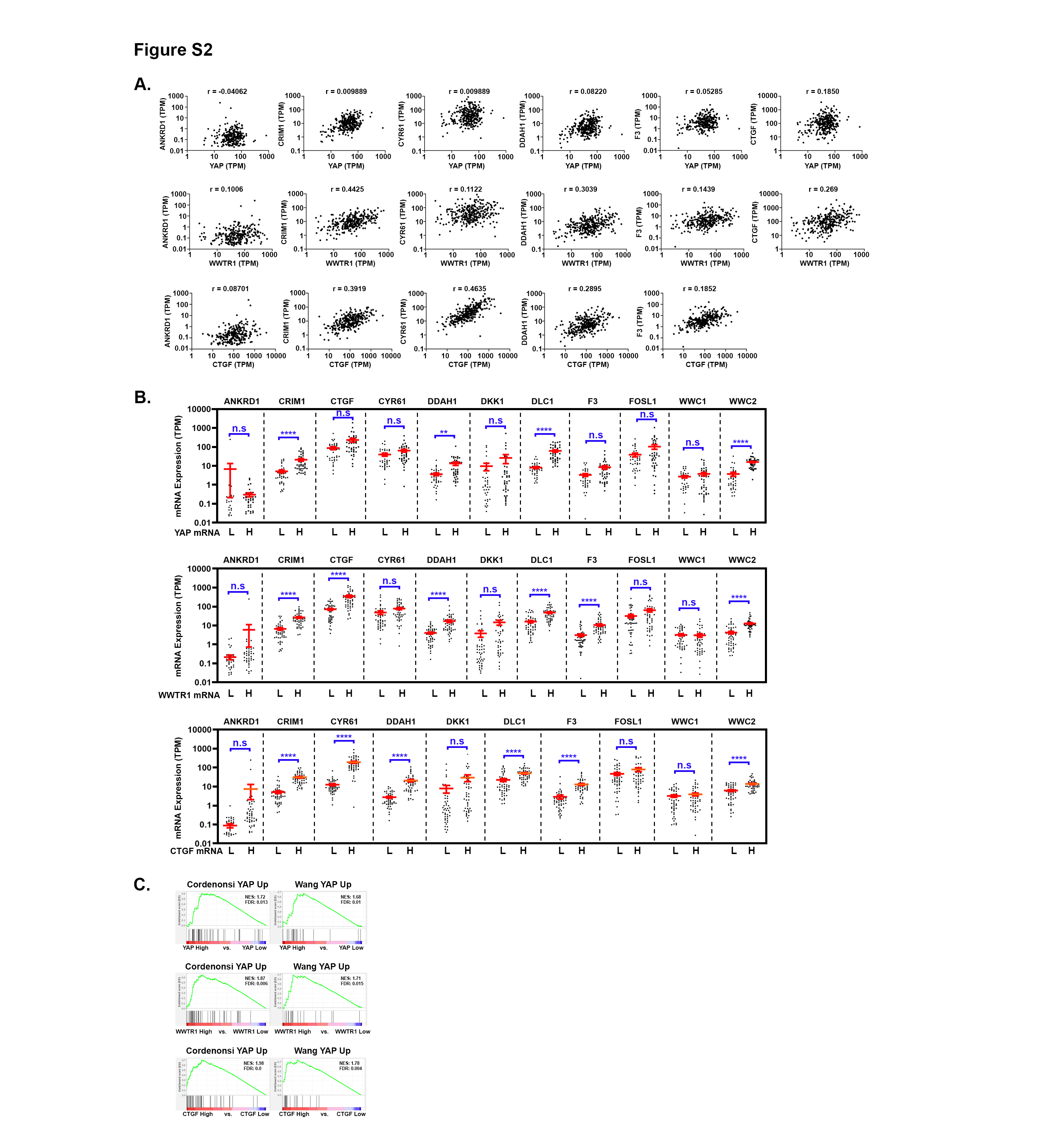

Supplement: Supplementary file 1 [file cancers-16-00852-s001.zip › Figure S2.tif]

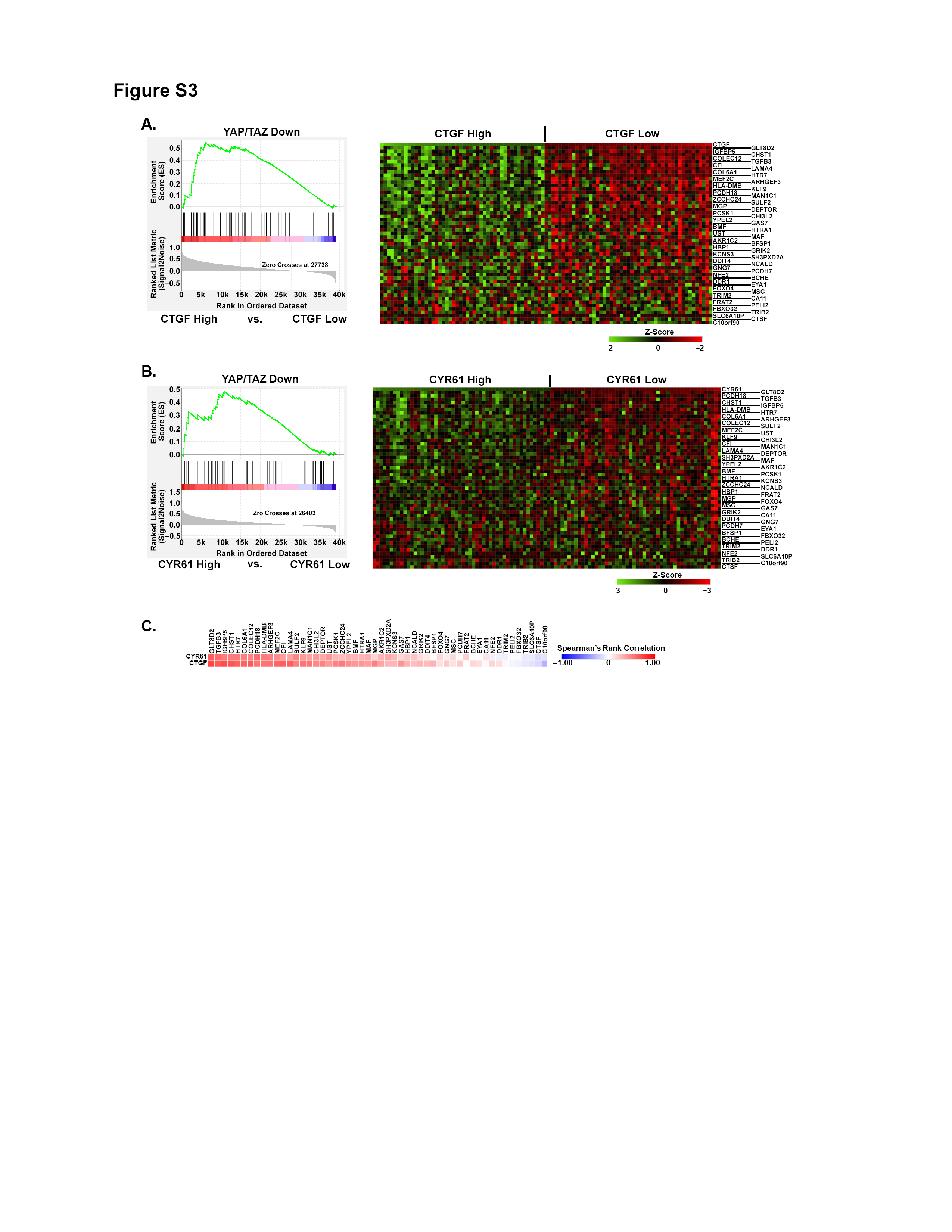

Supplement: Supplementary file 1 [file cancers-16-00852-s001.zip › Figure S3.tif]
